# The origins and implementation of an intervention to support healthcare staff to deliver compassionate care: exploring fidelity and adaptation in the transfer of Schwartz Center Rounds® from the United States to the United Kingdom

**DOI:** 10.1186/s12913-019-4311-y

**Published:** 2019-07-08

**Authors:** Mary Leamy, Ellie Reynolds, Glenn Robert, Cath Taylor, Jill Maben

**Affiliations:** 1 Department of Mental Health Nursing, Florence Nightingale School of Nursing, Midwifery and Palliative Care, King’s College London, James Clerk Maxwell Building, 57 Waterloo Road, London, SE1 8WA UK; 2 Adult Nursing Department, Florence Nightingale Faculty of Nursing, Midwifery and Palliative Care, King’s College London, London, UK; 3 School of Health Sciences, Faculty of Health and Medical Sciences, University of Surrey, Guildford, UK

**Keywords:** Schwartz center rounds®, Implementation, Fidelity, Staff wellbeing, Healthcare workforce, Compassionate care, Innovation

## Abstract

**Background:**

Schwartz Center Rounds® (henceforce Rounds) were developed in the United States (US) in 1995 to provide a regular, structured time and safe place for staff to meet to share the emotional, psychological and social challenges of working in healthcare. Rounds were adopted in the United Kingdom (UK) in 2009 and have been subsequently implemented in over 180 healthcare organisations. Using Rounds as a case study, we aim to inform current debates around maintaining fidelity when an intervention developed in one country is transferred and implemented in another.

**Methods:**

Interpretive design using nine qualitative interviews (UK = 3, US = 6) and four focus groups (UK: Focus group 1 (4 participants), Focus group 2 (5 participants; US: focus group 1 (5 participants) focus group 2 (2 participants) with participants involved in Rounds design and implementation, for example, programme architects, senior leaders, mentors and trainers. We also conducted non-participant observations of Rounds (UK = 42: USA = 2) and training days (UK = 2). Data were analysed using thematic analysis.

**Results:**

We identified four core and seven sub-core Rounds components, based upon the US design, and seven peripheral components, based on our US and UK fieldwork. We found high core component fidelity and examples of UK adaptations. We identified six strategies used to maintain high fidelity during Rounds transfer and implementation from the US to UK settings: i) having a legal contract between the two national bodies overseeing implementation, ii) requiring adopting UK healthcare organisations to sign a contract with the national body, iii) piloting the intervention in the UK context, iv) emphasising the credibility of the intervention, v) promoting and evaluating Rounds, and vi) providing implementation support and infrastructure.

**Conclusions:**

This study identifies how fidelity to the core components of a particular intervention was maintained during transfer from one country to another by identifying six strategies which participants argued had enhanced fidelity during transfer of Rounds to a different country, with contractual agreements and legitimacy of intervention sources key. Potential disadvantages include limitations to further innovation and adaptation.

**Electronic supplementary material:**

The online version of this article (10.1186/s12913-019-4311-y) contains supplementary material, which is available to authorized users.

## Background

Schwartz Center Rounds® (henceforth Rounds) provide a regular, structured time and safe place, for healthcare staff employed in all roles, functions, disciplines and grades (clinical and non-clinical), to come together to share the benefits and challenges of working in healthcare. The purpose of Rounds is to support staff and enhance their ability to provide compassionate care by providing a forum for reflecting and gaining insight into their own – and others’ – responses to the work they do. Unlike other types of reflective practice interventions, they are not a place for solving problems or focusing upon the clinical aspects of patient care, but for sharing the emotional, social and ethical challenges of providing care. In a scoping review, Taylor and colleagues have systematically searched for evidence on 11 interventions with similar aims of providing emotional support for healthcare staff with emotional challenges at work, and compared the effectiveness and key features to Rounds [1]. This paper suggests Rounds are unique in that they are open to all staff in an organisation, are on-going (not time-limited) and that there is no expectation to contribute.

Writing in his local paper, the Boston Globe, Kenneth Schwartz, wrote an article called “A patient’s story” about the care he received as a patient with advanced lung cancer [2]. He noticed that “*small acts of kindness made the unbearable bearable*” and how his “*ordeal has been punctuated by moments of exquisite compassion*”. When Kenneth Schwartz died, his close friends, family, nurses and oncology consultant, following his instructions about the type of organisation he wanted to see created, established the Schwartz Center for Compassionate Care (SCCC) (Schwartz, 1995). Over the last 24 years, Rounds have been adopted and sustained in North America by the SCCC and in the UK, via the (now) Point of Care Foundation (PoCF) [3] . In this study we use Paul Gilbert’s (2009) definition of Compassion: *“The motivation to be caring for the purpose of alleviating distress and facilitate the flourishing and development of the target of the caring. The skills required are sensitivity; sympathy; empathy; distress tolerance and being non-judgmental to create feelings of warmth, kindness and support” (p.203)* [4]*.*

In this paper we focus upon issues of fidelity - the extent to which an intervention as implemented later corresponds to the originally intended intervention (sometimes referred to as integrity) - and adaptation - the changes made to the original intervention by its implementers or users [5]. The fidelity versus adaptation dilemma is one of the major debates in implementation science research [6, 7]. Fidelity and adaptation are opposing concepts, although they are intrinsically linked. Broadly speaking, the fewer adaptations implementers make to an intervention the higher the level of fidelity, whereas the greater the adaption the higher the threat to fidelity and potentially to the intervention’s effectiveness, at least as originally conceived by the programme architects.

Carroll and colleagues have argued that although interventions cannot always be implemented as originally planned, they can still be meaningfully implemented if the essential components are retained [8]. In characterising complex interventions, some theorists have distinguished between the ‘hard-core’ elements of an intervention which are well-defined and fixed, and a ‘soft periphery’ that is vague and more flexible to manipulation by the adopting system or organisation [9, 10]. Denis and colleagues used this distinction to propose that the ‘negotiation of the meaning of an innovation in a particular context occurs in the soft periphery of its definition, enabling a variety of pathways to adoption’ [11]. In a similar vein, others have suggested interventions can be conceptualised as having ‘core components’ (the essential and indispensable elements of the intervention) and an ‘adaptable periphery’ (adaptable elements, structures, and systems related to the intervention and organisation into which it is being implemented) [9, 12, 13].

Transferring an intervention from one country to another is now commonplace. Yet Harris and colleagues have reported that the diffusion of innovation literature is silent on whether country of origin matters because it only focuses upon the characteristics of the adopter, not the source [14]. Damschroder and colleagues argue that the source of an intervention’s legitimacy is key, but they do not explain what that might mean for different intervention implementers or recipients [9]. Ferlie and Shortell highlight the importance of a country’s larger system infrastructure, for instance, national bodies, evidence-based practice centres, accrediting and licensing agencies, payment policies and legal systems in influencing fidelity and adaptation [15].

Using Rounds as a case study, we aim to inform the current implementation science debate around how best to maintain fidelity when an intervention developed in one country is transferred and implemented in another. Our four related objectives were:

1. To identify the origins and core components of Rounds in the US;
2. To identify strategies used to maintain high fidelity during Rounds transfer and implementation from the US to UK settings;
3. To identify and verify core-peripheral components of Rounds;
4. To assess fidelity and adaptation in the UK using the core-peripheral components.

## Methods

### Setting

Rounds have been implemented in the US (via the SCCC) from 1995, and in the UK (via the PoCF) from 2009. More than 440 healthcare organisations are currently running Rounds throughout the US, Australia, New Zealand and over 200 in the UK and Ireland [3].

### Study design

Interpretive design using qualitative interviews, focus group and observational data, formed part of a wider realist informed, mixed methods evaluation of Rounds in UK [16]. This wider study was designed to answer the research question: How, in which contexts and for whom, Rounds participation affects staff wellbeing at work, social support for staff and improved patient care. As only some of the methods used Realist Evaluation methodology (Pawson and Tilley) we have described out study as ‘realist informed’ [16].

### Data collection and analysis

To understand the origins and development of Rounds, we conducted fieldwork in the US and UK, which included a purposive and limited sample of key stakeholders interviews and focus groups with participants involved in designing and overseeing the implementation of Rounds (see Table 1).

**Table 1:** Summary of interviews, focus groups and observations

Country	Key stakeholder individual interviews	Key stakeholder focus groups	Observations
US	3 interviews (programme architects, lead SCCC facilitator)	2 focus groups, 7 participants in total Group. 1: Held at SCCC. SCCC programme architects, Director of programmes and Rounds training, Vice Chair of SCCC board, physician leaders and facilitators of Rounds at local hospitals (*N* = 5) Group 2: Physican lead/Schwartz Rounds facilitator (*N* = 2)	2 Rounds observed^a^
UK	6 interviews (PoCF senior leaders, trainers, lead mentor, programme manager, pilot site facilitators leads)	2 focus groups, 9 participants in total Both held in university venues Group 1: Mentors/ key PoCF stakeholders (*N* = 4) Group. 2: Mentors/ PoCF trainers (*N* = 5)	42 Rounds^b^ 2 PoCF facilitator training days

^a^No data collection undertaken, observed in capacity of external guest

^b^Data collected for wider UK evaluation of Rounds and reported elsewhere (Maben et al)

**Fig. 1:**
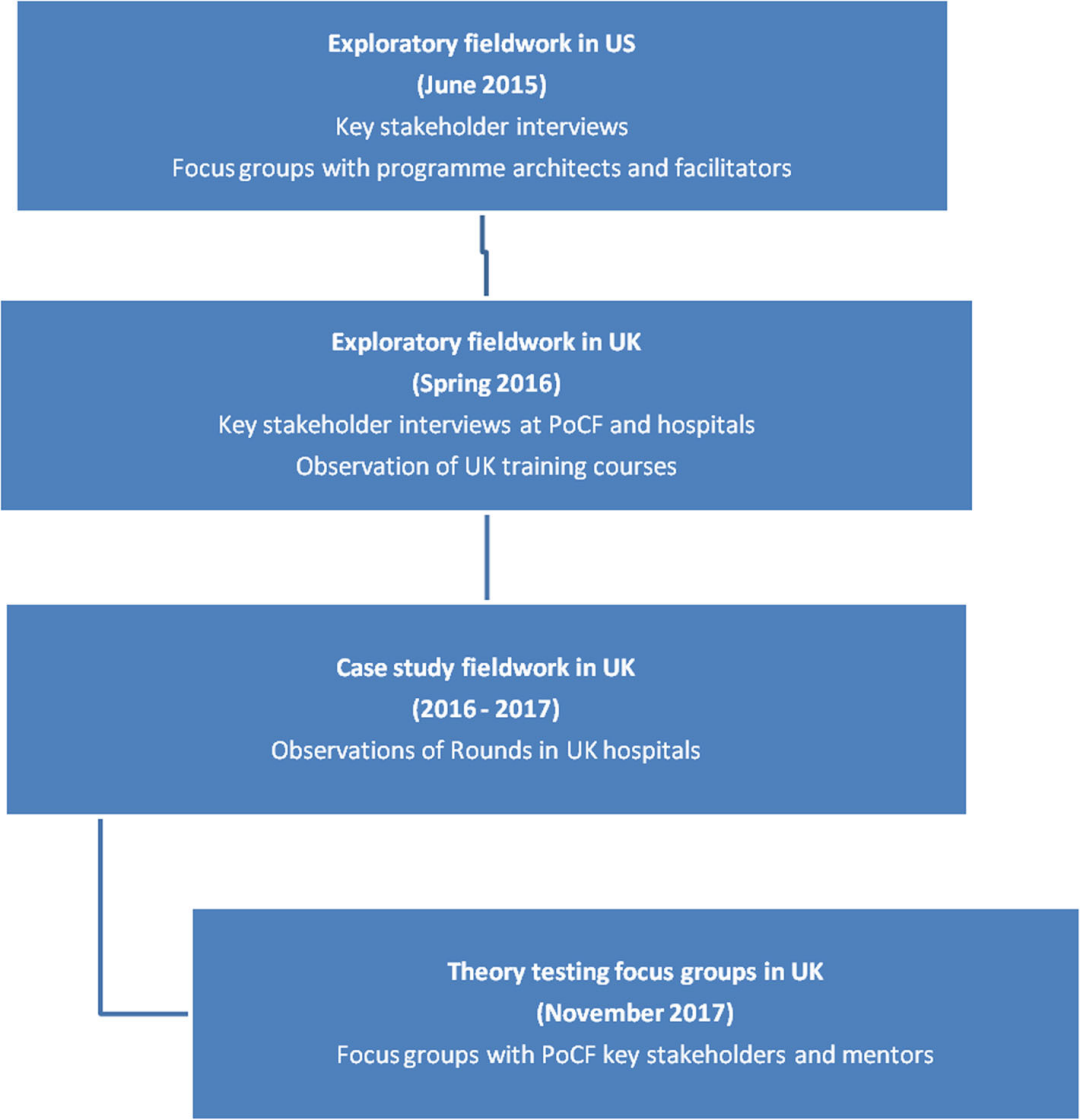
Timeline and flowchart of data collection

In 2015, two researchers visited the SCCC in US and conducted exploratory key stakeholder and focus group interviews. We asked the SCCC to provide contact details for key interviewees and identify regional Rounds being held during the study visit week. The interview schedule included history, rationale, development and spread of Rounds, implementation experiences, fidelity, quality assurance, and evaluating Rounds, and future plans. Key questions included: Can you tell me more about the history and thinking behind Schwartz Rounds? What impact do you think Rounds have on presenters and audience, wider organisation? How do you manage fidelity and quality assurance issues with USA and beyond? What lessons have you learnt about Schwartz Rounds? The focus groups explored implementation experiences, for instance supporting existing and new sites, training, and adaptations, including questions like: What USA contexts and settings have Rounds been tried in? Have you noticed differences in how Rounds work in different areas, settings, groups of people? If so, what are these differences? Can you describe the most memorable/most successful/unsuccessful Round you’ve been to and why was it memorable/ successful or unsuccessful? (See Additional file 1). On return to the UK, we conducted exploratory key stakeholder interviews with PoCF senior leaders, staff responsible for training and mentoring new facilitators and the pilot site facilitators who were involved in transferring Rounds to UK. Our interview topic guides were informed by the analysis of the US interviews/focus groups but also included the reasons for selecting Rounds as an intervention and issues regarding cross-cultural transfer to UK setting. All interviews and focus groups were conducted by two researchers (ER and ML in US; ML with ER or JM in UK). In the UK, focus groups with mentors and key PoCF stakeholders were held at the end of data collection and provided opportunity for theory-testing. We attended and observed US Rounds in the capacity of external guests, not as researchers. On return to UK an observation guide was developed for case study fieldwork observations (reported elsewhere, [16]. The timeline for US and UK data collection is illustrated in Fig. 1.

We identified a provisional list of ‘core’ components of Rounds (based upon the original US design) by concurrently examining the US contract and the data from our interviews with US programme architects to support and elaborate the contract. Having drawn up provisional lists of what appeared to be ‘core’ and ‘peripheral’ components, we then held focus groups to discuss these with Rounds mentors/experts in UK and refine descriptions of the core-peripheral components (see Results, Tables 3 and 4). We explained to participants that ‘peripheral’ meant components could be adapted without impacting significantly on the overall effectiveness of the intervention. We then re-examined our fieldwork data in the UK to assess fidelity and adaptation using these core-peripheral components. Interviews and focus groups were digitally recorded, professionally transcribed and analysed using an inductive thematic analysis approach, following key stages of data familiarisation, data reduction and interpretation [17]. The themes were examined across participants for consistency and, where appropriate, constructs were categorised into smaller units. The initial coding framework was developed by two researchers (ER and ML), who both analysed a sub-sample of transcripts separately, then met to discuss and compare coding, including areas of disagreement, and revise coding framework. Co-authors and the wider research team also meet frequently to reflect upon emerging findings during fieldwork and analysis. All data were coded in the qualitative data analysis software package NVIVO.

## Results

We present our findings to address objectives 1–4 in turn below.

### To identify the origins and core components of rounds in the US

The purpose of Rounds in the US was to demonstrate that compassion could be taught and improve connection and compassionate care for patients, achieved partly through role modelling certain kinds of behaviour, such as senior clinicians revealing their vulnerability. Rounds were expected to impact on patient experience through teaching compassionate care.

Rounds were designed to be multi-disciplinary, inclusive and provide a level playing field, because all staff face the same challenges in terms of connection and compassion.

*“We also knew that everybody touched the patient, because of Ken’s story. He talked about the technician that wheeled him down to the CT scan, that person showing compassion, so we knew that we wanted to include everybody in the hospital setting, this wasn’t just for doctors”. (Programme architect 1, US)*

Part of the specifics of their design and why Rounds were initially modelled on medical ‘Grand Rounds’ was to make them acceptable to doctors and to encourage their attendance. Subsequently this was formalized in the contract which required any organisation running Rounds to have senior medical staff taking a lead role:

*“I knew in the very beginning that I had to make them as medical as possible, so people would [not] see them as being soft, okay….in the first couple of Rounds, we actually presented X-rays and we talked about the case… Eventually we got away from presenting the X-rays and the medical stuff”. (Programme architect 1, US)*

Following the medical Grand Rounds format, Rounds had a topic, a panel and an audience discussion, but in Schwartz Rounds, unlike Grand Rounds, the focus became the non-medical aspects of care. One of the programme architects felt that their specific contribution had been to highlight the importance of the non-clinical aspects of care for caregivers and to find a format that would be acceptable to the medical profession. The space created in Rounds needed to be a safe environment to encourage people to speak and share their experiences.

*“You have to validate their opinion and realise that you’re creating a safe environment …so being non-judgmental, not allowing anyone to get bullied, is a huge part of Rounds”. (Programme architect 1, US)*

In the US, Rounds were co-facilitated, usually by a medical doctor and a second facilitator who could combine group moderation skills with a good understanding of psychology. Rounds needed to have visible support from the organisation’s leadership to make evident the organisation’s values on the importance of connecting with patients and supporting staff.

Based on the US-UK contract and our US interviews, the *provisional* list of core components, for US Schwartz Rounds were:

1. *Focus on emotional impact on staff* (e.g. vs. clinical details, problem-solving)
2. *Participants* (staff only vs. patients and staff)
3. *Leadership* (Senior medical leadership?)
4. *Facilitation* (e.g. group moderation skills, psychology knowledge)
5. *Safety* (e.g. Ground rules on confidentiality)
6. *Integrity* (e.g. not combined with other interventions, used for other organisational purposes?)
7. *Regularity* (i.e. not one-off events)
8. *Food* (available for Rounds attenders)
9. *Panellist preparation* (Guidance from facilitators to help prepare for Schwartz Round)
10. *Audience discussion* (Sufficient time for audience discussion)

These provisional core components, derived from the US Schwartz Rounds, were then verified with UK mentors/experts and refined during the study (see later).

### To identify strategies used to maintain high fidelity during rounds transfer and implementation from the US to UK settings

We identified six strategies which participants argued had enhanced fidelity during transfer of Rounds to a different country.

#### Legal contract between two national overseeing bodies

A legal contract outlined the nature of the relationship between the SCCC and King’s Fund. It specified that ‘*the Schwartz Center has a recognised and proven format for conducting successful Rounds, including educating the senior consultant/medical director and multidisciplinary planning group at each site on the purpose, format and benefits of Rounds, selecting a facilitator, identifying cases and panellists for presentations*’ (King’s Fund-SSSC, 2009). The PoC programme paid SCCC a one-off partnership fee to recognise the SCCC’s work in developing and establishing Rounds, and an annual fee to use the Schwartz Rounds® name, intellectual property and for SCCC staff time to assist the PoC programme to implement Rounds.

#### Use of contracts between the national overseeing body and healthcare organisations

The US-UK contract required the PoC programme to enter into separate contacts with individual healthcare organisations to fulfill the requirements regarding maintenance of the ‘*general Rounds’ format’*, *integrity* and *regularity*, stating that any adaptations required written agreement. It is noteworthy that the PoCF have decided to adopt a light touch approach to monitoring implementation. Whilst they require sites to evaluate each Round using participant feedback forms and send these back to PoCF, and visit sites to provide ongoing support and some measure of oversight, monitoring Rounds providers in a meaningful way is reported as challenging. In situations where healthcare organisations have not complied with the contract, the PoCF have formally reminded them of their obligations under the contract, be sending a letter to the chief executive, as a way of supporting the Trust’s facilitators. When asked if there were any circumstances where they would terminate the contract, they felt it was possible they would, though this has not happened to date.

*“The answer is we don’t know yet really….if they were going really off piste I think we would probably say ‘enough’ … they [SCCC] have done that on a very small number of occasions*”. *(PoCF senior leader 1, UK)*

#### Piloting the intervention in the UK context

In 2009–10, the pilot implementation of Rounds in two UK hospitals was evaluated [18], primarily to test the feasibility and acceptability of Rounds in a UK context and the extent to which Rounds had similar outcomes as those reported in the US evaluation [19]. The evaluation used participant post-round evaluation forms, a pre-post online survey of Rounds participants using the same questionnaires as the US study, and qualitative interviews with key staff involved in establishing and running Rounds in both trusts, at the start and end of the piloting period [18]. The UK pilot study findings replicated the outcomes found in the US evaluation, with participants reporting Rounds had supported their delivery of patient care, improved team work and made a positive contribution to organisational culture. On the basis of the pilot study findings that Rounds were feasible and acceptable in a UK context, the Rounds were implemented more widely in England.

#### Using credibility of intervention attribution and of adopter sources

The origins of Rounds are directly attributable to Kenneth Schwartz’s personal experience of compassionate care and his oncology nurse’s suggestion that staff need to have a place to talk about the emotional cost and personal impact of caring. In both countries, we observed Rounds often starting with a synopsis of Kenneth Schwartz’s story, to maintain and emphasise the ‘source legitimacy’ of the intervention (ref Damschroder and colleagues). One of the PoCF senior leaders also felt the excellent reputation of the King’s Fund as a thought leader in UK healthcare had a significant role in persuading the SCCC to transfer responsibility for UK implementation of Rounds.

#### Promoting and evaluating Rounds

As well as sharing the ‘*generalised Rounds format*’, the SCCC also shared their proven strategies for persuading organisations about the purpose, benefits and key features of Rounds, and strategies for supporting implementation of Rounds at new sites to ensure fidelity. In promoting Rounds, the PoCF drew upon the evidence provided by the US and UK evaluations for the value and contribution of Rounds, [18, 19], wider research linking staff and patient wellbeing [20, 21], despite at the time, this being a relatively limited evidence base. In the UK, Rounds adoption was also linked to the Francis report [22].

#### Infrastructure and support

It is noteworthy that initially in the USA, the SCCC was covering all the costs of running Rounds in hospitals. Whilst they did not train facilitators and clinical leads, they did pay the facilitators and supplied them, and provided grants for the costs of the catering. This gave the SCCC a degree of control over the product that the PoCF in the UK could not afford. Over time, the PoCF developed its own infrastructure for Rounds. One of the pilot site facilitators became lead mentor and trainer for the PoCF and developed a handbook, a training workshop and nationwide mentoring scheme (*n* = 20 mentors) (from 2013). This infrastructure and support served to standardise aspects of Rounds structure and delivery in the UK, much more so than in the US. As part of the US-UK legal agreement to share any ‘*discoveries, improvements or other intellectual property conceived or reduced to practice’* (King’s Fund-SSSC, 2009), these developments were shared and subsequently have been adopted in the US. In practice this has meant that the adopter country influenced the implementation strategies in the originator country. Most recently, one of the facilitators from the UK Trust that introduced ‘pop-up’ Rounds (shorter Rounds taken to ward-based teams) was invited by the SCCC to their annual Schwartz Rounds conference in Boston to talk about them.

### To identify and verify core and adaptable peripheral components of rounds

The provisional list of ‘peripheral’ components was identified through our observation of Rounds variability and interviews in US and UK:

1. *Diversity* (e.g. open to all vs. targeted? multi-disciplinary vs. uni-disciplinary?)
2. *Panellists* (is there a minimum number?)
3. *Staff stories* (live vs. filmed stories)
4. *Duration* (1 hour vs. less than 1 hour?)
5. *Scale* (single organisation vs. multi-organisations)
6. *Format* (live vs. teleconferencing/videoconferencing; panellists stories first vs. staff stories and audience discussion integrated)
7. *Frequency* (more vs. less than monthly).

Following discussions within the UK Rounds mentor focus groups and research team, this was reduced to four, as seven components were subsumed within a new, broader *‘Generalised Rounds format’* component and became sub-core components (see Table 2). In addition, the descriptor for *‘Rounds leadership’* was expanded to include the contribution of the multi-disciplinary planning group and need for support from Trust board and Chief Executive.

**Table 2:** Rounds fidelity: core components following UK focus groups

1. Focus and purpose	“*Focus is the social and emotional issues that arise in caring for patients”* and *“differ from typical clinical Rounds”* (e.g. not problem-solving, outcomes-oriented, clinical focus). Purpose is to support healthcare workers deliver compassionate care.
2. Generalised Rounds format	To share experiences around a theme or case, to trigger reflection and audience discussion. ‘*Generalised Rounds form*at’ includes:
2a. Co-facilitation	Senior consultant/medical director and facilitator with group moderation skills and knowledge of psychology.
2b. Pre-prepared staff’ stories	Guidance on crafting story and identifying what will resonate with audience.
2c. Audience discussion	Sufficient time for audience discussion
2d. Participants	Rounds are open to everybody (e.g. multi-disciplinary and inclusive), and includes a distinction between panellists and audience
2e. Safe environment	Need for ground rules on confidentiality, and facilitators to create a supportive, non-judgemental safe space
2f. Rounds Leadership and visible organisational support	“*Senior consultant/medical director”*, “*multi-disciplinary planning group*” and “*ensure support of Trust board and Chief Executive*”.
2g. Food	*Organisations will be responsible for providing food Rounds for attenders*
3. Integrity	Rounds ‘*not combined with other clinical Rounds or any other program’*
4. Regularity	A series of events over time, i.e. not one-off events

*italics are taken from clauses in US-UK contracts, otherwise they come from US fieldwork data

This table is reproduced with permission from NIHR

The peripheral components were also revised in discussion within the UK Rounds mentor focus groups and research team. The ‘*Regularity*’ component descriptor (the requirement that Rounds were held on a monthly basis) was modified to accommodate UK contextual differences so that the number of Rounds per year was linked to the size of an organization. Unlike in the US, the UK run Rounds in hospices. Whilst focus group participants agreed the ‘*Number of panellist*s’ component could be varied, they specified parameters because they believed that fewer than two panellists would potentially impact upon the general format [2] and levels of safety (2e), and having more than four panellists would reduce time for wider audience discussion (2c) (see Table 3).

**Table 3:** Rounds fidelity: peripheral components following UK focus groups

1. Diversity	Rounds can be targeted to specific groups of staff
2. Number of panellists	Can vary, but within parameters (e.g. minimum two, maximum four)
3. Type of Rounds	Theme-based Rounds (panellists’ stories related by a theme, but about different patients), Case-based Rounds (all panellists speaking about caring for same patient) and Patient-presenter Rounds (mixed panel of staff and patient/s).
4. Duration	Can vary, but within parameters (e.g. minimum half an hour, maximum one hour)
5. Scale	Can vary, e.g. specific function within an organization, be organisation-wide, or involve multiple organizations within a locality
6. Generalised Rounds Format	Use of technology such as teleconferencing, videoconferencing
7. Frequency	Determined by organisational size (large sites should run at least nine Rounds per year and smaller sites/hospices four per year).

This table has been reproduced with permission from NIHR

### To assess fidelity and adaptation in the UK using the core-peripheral components

Using the core-peripheral component distinction to revisit fidelity and adaptation that we had observed in the 42 UK Rounds (as set out in see Tables 2 and 3), we found that core component fidelity had largely been maintained (see Table 4 below).

**Table 4:** Implementation of core-peripheral components in UK organisations

Core components		
Component	As implemented in practice in UK	Fidelity/adaptation
1: Focus and purpose	Focus on social and emotional issues for staff maintained by skilled facilitation Implementers conceptualise Rounds primarily as a staff wellbeing intervention, which then links to improved patient experiences of care [2]	High fidelity to focus Some adaptation to purpose
2: Generalised Rounds format	Formally structured, tight control over format Clear distinction between panellists and audience	High fidelity
2a: Co-facilitation	Facilitators come from a range of backgrounds. Minority of organisations only have one facilitator	Usually high fidelity, some adaptation
2b. Pre-prepared staff stories	The extent and nature of preparation varied between Rounds sites and facilitators-some phone only others face to face. Occasionally panellists given virtually no preparation.	Usually high fidelity
2c. Audience discussion	Audience discussion time varied, but usually between 30 and 40 min	High fidelity
2d. Participants	Rounds open to all staff; Medical professions attendance is encouraged, but not crucial. Majority of Rounds have at least one doctor present, others many doctors present. Clear distinction between panellists and audience.	High fidelity
2e: Safe environment	Pre-Round emotional and psychological ‘safety checks’ during panel preparation; Confidentiality sign-in form and ground rules and facilitators support contributors to feel safe.	High fidelity
2f: Rounds Leadership/organisational support	Rounds sites often have ‘Medical’ leads, though some sites are led by other disciplines, i.e. ‘Clinical’ leads Board/ senior managers presenting and/or attending Rounds All sites have multi-disciplinary planning groups, though participation and attendance vary	Usually high fidelity, some adaptation
2g: Food	All Rounds have food provided. Some sites provide cold buffet, others provide hot dishes.	High fidelity
3. Integrity	Educational aspects present but not explicitly emphasised (e.g. Role modelling/ discussions of excellent practice). Rounds not combined with other interventions.	High fidelity
4: Regularity	All organisations run Rounds as ongoing events, rather than one off. Some sites have runone-off ‘demonstration Rounds’ to publicise them.	High fidelity
Peripheral components		
Component	As implemented in practice in UK	
1. Diversity	Rounds targeting single professions, specific wards or specialty based and only ran in part of an organisation. Usually adapted Rounds for specific staff groups *are held in addition* to Rounds which are organisation-wide.	
2: Number of panellists	Usually Rounds have three or four panellists. One site always ran Rounds with a single ‘presenter’, but found they were unable to sustain Rounds within the organisation because of lack of willing ‘presenters’.	
3: Type of Rounds	UK sites only have ‘theme’ or ‘case’ based Rounds. Not running Patient-presenter Rounds as mentors and trainers believe that having a patient present at Rounds alters the group dynamics and purpose.	
4. Duration	‘Pop up’ Rounds are small scale Rounds. They only last half an hour and are offered in addition to organisation-wide Rounds. They are designed to reach staff who cannot usually attend Rounds (e.g. ward-based staff).	
5: Scale	Scaled down (e.g ‘Pop up’ Rounds). Scaled up (e.g. participants from other healthcare organisations).	
6: Generalised Rounds format	Some experimenting with format to hold Rounds which use pre-recorded films to stimulate discussion, or invite panellists/ audience to attend via teleconferencing/ videoconferencing.	
7. Frequency	Rounds are usually monthly, except for peak holiday periods (e.g. December and August). Some cancellations due to last minute panellist drop out, low audience numbers. A couple of examples of large healthcare organisations holding two or more Rounds a month, at different hospital sites, or rotating each month between sites.	

When looking at UK implementation, we found examples of where the “*soft periphery*” of Rounds had been adapted to increase reach, participation and sustain Rounds, which led to different pathways to adoption. Smaller scale ‘Pop up’ Rounds, which were designed to enable ward-based nursing staff to attend Rounds, had adapted more than one peripheral component, namely ‘*duration*’ and ‘*scale*’. By only having one facilitator however, ‘Pop up’ Rounds had also lowered fidelity to the ‘*co-facilitation’* core component and because they were offered to particular staff groups multi-disciplinarity may be compromised in some settings and as a one-off they lowered fidelity to the ‘*regularity*’ core component. Rounds in one site only had one ‘presenter’ per Round, which was reportedly often hard to facilitate and we noted how distinctly different it felt to any other Schwartz Round. We concluded this led to less meaningful implementation as a result of this adaptation, a view that was subsequently confirmed by our focus group participants. Other ways in which the soft periphery components had been adapted included using teleconferencing and videoconferencing technology instead of holding Rounds as face-face events, and using pre-recorded films of staff stories to trigger audience discussion. These types of adaptations have arisen to address problems created when organisations have geographically dispersed sites and when panelists drop out at short notice. Table 4 presents the final list of core and peripheral components, after verification with UK mentors/experts.

## Discussion

Rounds are now running in other countries, but the UK was the first country to implement them outside of the US and demonstrate that they could be transferred into a different healthcare system, with similar outcomes, initially through a pilot evaluation [18], and subsequently in the wider evaluation study which this paper relates to [16]. To address the question of whether country of origin matters we have identified four important aspects: i) the extent to which a country’s larger system infrastructure influences fidelity and adaptation [14, 15], ii) the legimacy of an intervention’s source, iii) the degree to which core components are defined and retained, and iv) the specific use of reproducible implementation strategies.

Firstly, in terms of infrastructure, we note the importance of having two reputable national charitable bodies (SCCC and King’s Fund) to oversee Rounds implementation. These organisations were instrumental in developing arrangements for providing training, mentoring support and ‘Schwartz community’ networks. Secondly, we would support Damschroder and colleagues’ view that the source of an intervention’s legitimacy is key [9]. Rounds had been inspired by the healthcare experiences of Kenneth Schwartz whilst he was a patient, and developed by medical and nursing staff that had cared for him, and his close family and friends. At the start of each Round UK participants are regularly reminded of these origins which help to maintain and emphasise this ‘source legitimacy’.

Thirdly, the extent to which core components are defined and retained; from our data, we have concluded that even when an intervention cannot be implemented as originally planned and when the country of origin is different from the country of implementation, an intervention can still be meaningfully implemented if the essential components are retained. Schwartz Rounds achieved this by ensuring the majority of the “hard-core” components were well-defined and fixed and specifically use reproducible implementation strategies, as set out in a US-UK legal contract, with detrimental consequences if the contract was not enforced (e.g. the PoCF would lose their licence to run Schwartz Rounds in the UK). One exception to this was the ‘*generalised Rounds format*’ core component. Our data helped us to unpack and describe what ‘*generalised Rounds format’* (as cited in the contract) comprised of and separate it into sub-core components. Finally, Proctor and colleges [23] propose implementation strategies which need to be adequately operationalised: the actors(s); the action(s); the target(s) of the action; temporality; dose; the implementation outcome(s) affected; and justification. Some of these implementation strategies were built into the US-UK contract (e.g. who facilitates, who participates, the purpose and justification for Rounds). However, we noted that the degree of specification was at times insufficient to fully operationalise the implementation strategies.

### Limitations

The main limitation of this study is that whilst we observed and collected data from 42 Rounds in UK, we were not able to collect data from a similar amount of Rounds in the USA, which prevented us from exploring Rounds fidelity and adaptation to core-peripheral components in the US.

Whilst the ‘peripheral’ component adaptations were discussed and verifed with UK Rounds mentors/experts, who thought that these components would not have significant impact upon effectiveness of Rounds, they have not been rigorously evaluated. It is not known whether the ‘peripheral’ component adaptations may also have unintended impact on other ‘core’ components (e.g. safe environment) or Rounds outcomes and work has yet to be undertaken to identify and verify peripheral components in the USA.

### Implications of findings

This case study illustrates the benefits of having clarity about the core components formally set out in a legal contract between countries and contracts with individual healthcare organisations as a strategy to maintain fidelity. At a recent implementation science conference, Schwarz and colleagues called for researchers to put more emphasis on identifying core intervention and implementation strategy components. Making the core-peripheral components explicit helps practitioners and those implementing interventions adapt interventions without sacrificing meaningful fidelity [24]. Without such clarity there is a danger that adaptations or reinvention can impact upon the quality of care and outcomes associated with an intervention. The subsequent conference discussion highlighted that often, the question is not a matter of either fidelity or adaptation, but rather having a continuum of fidelity *and* adaptation. Whilst we would agree that identifying and verifying the core and peripheral components of an intervention is valuable activity, specifying what is ‘essential and indispensable’ is still open to interpretation and may not be as clear cut in practice. All Rounds experts agreed that providing food is fundamental to the format, (because it helps organisations demonstrate they value and respect their staff, and contributes towards creating a supportive and counter-cultural space) [16], but it is debatable whether this core component should hold the same weight as the ‘*safe environment’* core component. In addition, as our research has shown, components are not always mutually exclusive, so any adaptation to one core or peripheral component can inadvertently impact upon others, making evaluation harder because they cannot easily be isolated from one another. For example, in the UK, we occasionally observed Rounds with a single facilitator (‘*Co-facilitation’* core component) which had the potential to also impact on the level of safety (‘*Safe environment’* core component). In our wider study we report that Rounds safety is dependent upon many factors, for example Rounds topic/theme, facilitator skill and experience and audience familiarity with Rounds [16]. In sum, we have concluded that there are two important gaps in the implementation science literature, both relating to whether the distinction between core and peripheral components is really as black and white in the real world. Firstly, our findings illustrate the inter-connectivity of ‘core’ and ‘peripheral’ components and show how because of this inter-connectivity, adaptations to one component can have unintended consequences to others. Secondly, we would argue that the classification of what is a ‘core’ component may need to be more nuanced.

Whilst we would argue that the US-UK contract was central to this success, there were some potential disadvantages. Just having a legal contract with specified components may limit innovation. Further, whilst the US-UK contract clearly outlines how they should be implemented and monitored, the PoCF made a conscious choice to adopt a light touch approach to monitoring which in practice may mean fidelity is compromised. Without a mechanism to ensure fidelity is monitored, an organisation that does not follow the terms of the contract or engage in training, mentoring and networking events and activities, may drift away from the original intervention’s purpose and design. There is a potential trade-off between fidelity and innovation. Without the US-UK contract, we cannot know what may have happened, but we observed and heard about more examples of innovation and willingness to adapt Rounds in US than in the UK, potentially suggesting there may have been even more innovation and adaptation in the UK without the contract. As outlined above developments and innovation from the UK have been shared and subsequently adopted in the US (national training and pop-up Rounds for example), thus the adopter country (UK) has influenced the implementation strategies in the originator country (US).

More detailed organisational and clinical practice implications arising from the wider study evaluating Rounds is available elsewhere [16].

## Conclusion

There is no shortage of examples of healthcare innovations being adopted from other countries, but the literature on practical ways of maintaining fidelity during these transfers between countries is limited. This article has highlighted some of these practical measures the Schwartz Rounds implementers have taken to maintain fidelity during transfer from one country to another, with contractual agreements and the original source of the intervention playing a central role. We have also identified two gaps in the implementation science literature and questioned whether the distinction between core and peripheral components is as clear cut in reality. Potential disadvantages include limitations to further innovation and adaptation.

## Endnotes

The Schwartz Center for Compassionate Care, US is an autonomous, nonprofit organisation and relies on tax-deductible charitable contributions from foundations, corporations and individuals to carry out its work.

The Point of Care Foundation was established in 2013 as an independent charity. Prior to this it was known as the Point of Care programme which was established in 2007 and was hosted at the King’s Fund.

Medical Grand Rounds are formal meetings for doctors to discuss the clinical case of one or more patients. Grand Rounds originated as part of medical education training as a way of teaching new information and enhancing clinical reasoning skills.

## Additional file

Additional file 1: US interview/focus group schedule. (DOCX 19 kb)

## Data Availability

The datasets during and/or analysed during the current study available from the corresponding author on reasonable request.

## Abbreviations

PoCF: Point of Care Foundation
SCCC: Schwartz Centre for Compassionate Care
UK: United Kingdom
USA: United States of America
