# Supplementary material for: The origins and implementation of an intervention to support healthcare staff to deliver compassionate care: exploring fidelity and adaptation in the transfer of Schwartz Center Rounds® from the United States to the United Kingdom
Source: BMC Health Serv Res. 2019 Jul 8;19:457. doi: 10.1186/s12913-019-4311-y (PMC6615238; doi:10.1186/s12913-019-4311-y)
Supplement: Supplementary file 1 — US interview/focus group schedule. (DOCX 19 kb) [file 12913_2019_4311_MOESM1_ESM.docx]

**Supplementary file 1: Interview /focus group schedule**

1. **Origins of Schwartz Rounds**

- Can you tell me more about the history and thinking behind Schwartz Rounds?

Prompts:

- Were the Schwartz Rounds modelled on an existing intervention, if so, what?
- What was the underlying thinking behind Schwartz Rounds?
- What were the initial thoughts and underlying assumptions behind Schwartz Rounds?
- Why were Rounds needed?
- How did you anticipate they would work? Has this view changed?
- What kinds of impacts it would have upon staff and patient care?
- Who would benefit? What settings it would be most useful in?

1. **Current thinking behind Schwartz Rounds/ USA experiences (CMOs)**

- What elements did you consider important when designing Rounds to make them work?

Prompts:

- What ideas can you think of that explain how SR work?

1. **What impact do you think Rounds have on presenters and audience, wider organisation?** Prompts:

- Intended, unintended consequences? Any surprises?

1. **What USA contexts/settings have they been tried in? (Contexts)**

Prompts:

- Geographical areas/states? Types of organisation? Groups of participants?
- Have you noticed differences in how Rounds work in different areas, settings, groups of people? If so, what are these differences?
- What lessons have been learnt from USA Schwartz Rounds?
- How have they been adapted to suit different settings/professional groups/types of organisation? Which contexts do they work best in and why? What settings are Schwartz Rounds best least suited to and why? What groups of professionals are Schwartz Rounds best/least suited to, and why?
- What communication networks, both formal and informal, have developed between Rounds providers in the States, if any?
- Have you made any changes to the way Rounds are run following their implementation in the UK?

1. **Fidelity and quality assurance**

- How do you manage fidelity and quality assurance issues with USA and beyond?

1. **Spread of Rounds**

- How and where have you publicised Rounds more widely?
- What other countries have adopted/will be adopting them?
- Do you have any data on the uptake of Rounds and is it related to any specific incidents?
- Do you have a strategy for spreading Rounds? If so, what?

1. **How are you monitoring and evaluating SR? (Evaluation)**
2. **Own experience of Rounds**

- Can you describe the most memorable/most successful Round you’ve been to?

Prompts:

- Why was it memorable/ successful?
- How do you manage to keep coming up with ideas for presentation?

1. **Have there been any Rounds which you’ve thought did not really work?**

- Prompts:
- Tell me about that/those Rounds? If what way/s did they not work?
- How and why did they differ from other Rounds you’ve attended?

1. **General observations**

- What did you see as the advantages and disadvantages of Schwartz Rounds?
- What lessons have you learnt about Schwartz Rounds?
- From your experience, what would be the three top tips you would give someone wishing to start Schwartz Rounds in their organisation?

1. **Future plans**

- What are your future plans for Schwartz Rounds?

1. **And finally**

- Is there anything else you would like to say?
